# Graves Disease is Associated with Increased Risk of Clinical Alzheimer’s Disease: Evidence from the Medicare System

**DOI:** 10.21203/rs.3.rs-2596630/v1

**Published:** 2023-10-12

**Authors:** Arseniy Pavlovich Yashkin, Stanislav Kolpakov, Svetlana Ukraintseva, Anatoliy Yashin, Igor Akushevich

**Affiliations:** Duke University Social Science Research Institute; Duke University; Duke University; Duke University; Duke University

**Keywords:** Graves Disease, Alzheimer’s Disease, hyperthyroidism, dementia, Medicare

## Abstract

**Background:**

Identification of modifiable risk factors for Alzheimer’s Disease (AD) onset is an important aspect of controlling the burden imposed by this disease on an increasing number of older U.S. adults. Graves disease (GD), the most common cause of hyperthyroidism in the U.S., has been hypothesized to be associated with increased AD risk, but there is no consensus. In this study, we explore the link between GD and risk of clinical AD.

**Methods:**

Cox and Fine-Grey models were applied to a retrospective propensity-score-matched cohort of 15,505 individuals with GD drawn from a nationally representative 5% sample of U.S. Medicare beneficiaries age 65 + over the 1991–2017 period.

**Results:**

Results showed that the presence of GD was associated with a higher risk of AD (Hazard Ratio [HR]:1.15; 95% Confidence Interval [CI]:1.07–1.23). Magnitude of associated risk varied across subgroups: Males (HR:1.19; CI:1.01–1.41), Females (HR:1.09; CI:1.02–1.18), Whites (HR:1.13; CI:1.04–1.20), Blacks (HR:1.33; CI:1.04–1.20). Competing risk estimates were consistent with these findings.

**Conclusions:**

A potential mechanism connecting GD and AD may involve shared etiological factors between the two diseases. Although replication of our findings is needed, they suggest that GD prevention and treatment may contribute to reducing the burden of AD in U.S. older adults.

## 1.0. Background

Alzheimer’s disease (AD) is a debilitating neurodegenerative disorder, most prevalent in the elderly. Indeed, although AD is not part of the natural aging process, age is the most important non-genetic risk factor for the onset of this condition. AD places great strain on the U.S. healthcare system(1, 2) as well as on the health and financial well-being of family members and informal caregivers(1, 3–8). At this time there is no clinically validated treatment available for AD other than palliative care. A potential new pharmacological treatment has recently been introduced. However, the long term effects this may have on public health are unclear as there are still questions about the efficacy of this agent in slowing and/or reversing the neurodegenerative process associated with AD(9–12). Even if effective, this treatment would not remove AD-related financial burdens as it comes with significant monetary cost(13, 14). In the future, the prevalence proportion of AD is expected to increase, as is the size of the pool of at-risk individuals (15–18); therefore, significant attention has been placed on prevention efforts and the identification of modifiable risk factors associated with the onset of AD.

Graves disease (GD) is an autoimmune disorder that causes overproduction of thyroid hormones which can accelerate the metabolism, leading to a weight loss, rapid heartbeat, and other symptoms; GD is the most common cause of hyperthyroidism in the U.S. (19). Current estimates of the prevalence of GD in the U.S. range between 20 and 50 per 100,000 (20) with women (approximately 40 per 100,000) being at higher risk than men (approximately 10 per 100,000) (19, 21). Unlike AD, GD can be treated successfully. However, short of total thyroidectomy or equivalent non-surgical intervention(22), there is no permanent cure, with other treatments associated with high rates of recurrence and low rates of disease remission (19, 22, 23). Although GD is not an aging-related disease *per se*, with incidence peaking between 30 and 50 years of age(19), some complications of GD are more common in the elderly (19, 24). Increased risk of cognitive decline and the onset of AD/dementia have recently been associated with hyperthyroidism and GD (25–31). If correct, then mitigation of the risk associated with GD, through either prevention and/or successful treatment, becomes an actionable policy target with clear benefits both directly to individuals with hyperthyroidism and indirectly to the public by reducing the magnitude of the burden associated with AD. In this study, we will explore the potential relationship between GD and the risk of clinical AD/dementia in later life through a comparison of propensity-score matched groups of individuals drawn from a nationally representative sample of U.S. individuals age 65+.

## 2.0. Data and Methods

Data came from a nationally representative 5% sample of Medicare beneficiaries provided by the U.S. Centers for Medicare and Medicaid Services (CMS). In the U.S., the Medicare social health insurance system pays for the healthcare of over 98% of the U.S. population age 65+. The dataset spanned the 1991–2017 time-period and provided individual-level information on the dates of birth and death (if applicable), race, gender, and the diagnoses made (using International Classification of Diseases 9th (ICD-9) and 10th (ICD-10) revision codes) during episodes of care paid for by Medicare Parts A (facility) or B (professional) over that period. We limited our analysis to individuals living within the U.S. and enrolled in either traditional fee-for-service Medicare or a Medicare Advantage plan whose claims are processed by the CMS. Most Medicare Advantage plans do not share claims data with the CMS and therefore information on their beneficiaries is not available for research.

For the calculation of trends in age-adjusted incidence and prevalence, we required the beneficiary to be age 65 + and aggregated all individuals older than 100 into the 100 + age group (this was done both to simplify the age-adjusting process and to better comply with data reporting restrictions set forth by the CMS). The resulting samples ranged from 1,649,134 individuals in 1994 to 1,826,186 individuals in 2017. Although estimates for 1991–1993 are provided, these should be taken with care as many individuals in this period are still in the process of accumulating diagnoses after entry into the data. Incidence was calculated as the number of new cases of GD diagnosed before the end of a given year divided by the number of at-risk individuals present during that year. Prevalence was calculated as the number of living individuals diagnosed with GD on or before the end of a given year divided by the total number of living individuals. Age adjustment was done using the U.S. population for the year 2000 provided by the U.S. Census Bureau.

For the survival analysis portion of the study, we additionally required the presence of at least 3 years of look-back to ascertain the presence of baseline comorbidities, and at least one year of follow-up. The baseline age was set at the time of a verified GD diagnosis for the cases and three years after data entry for the controls. The look-back period was measured both from the individual age and from the calendar time perspective, making the minimum baseline age about 67.5 and the minimum baseline time January 01, 1994. Individuals with AD on or before the baseline date were excluded. The final sample pool for the survival analysis portion of the study consisted of 3,109,022 individuals.

The presence of co-morbid conditions from the Elixhauser co-morbidity index index(32), Graves Disease (ICD-9:242.00, 242.01; ICD-10:E05.00, E05.01), and Alzheimer’s Disease (ICD-9:331.0; ICD-10:G30), our primary outcome, were identified as follows: For AD, and GD we required at least two distinct claims no more than two years from each other. Date of onset was set at the earliest date of the two. This was motivated by the relative rarity of the conditions, and the need to mitigate the potential bias associated with erroneous diagnosis. Studies on GD relying on biologic data(33), have required the presence of two distinct serum thyroid-stimulating hormone concentration test values of < 0.3 mIU/L. Although our data does not have access to test results (therefore we do not observe if the < 0.3 mIU/L cut-off was reached), requiring a second episode of care with GD recorded as a diagnosis helps to approximate this process. For the Elixhauser-based co-morbidity index a more standard requirement of 90 days between two distinct claims was used. We also included the following socio-demographic covariates: male gender; Black, Hispanic, and Other (including Asians, Native Americans/pacific islanders), race, dual eligibility (as a proxy for poor economic status) and a yearly trend (to represent changes in technology and practice).

Of the 3,109,022 individuals eligible for the analysis, 17,505 were identified as GD cases. After comparing the summary statistics between these groups, we concluded that the GD group was too dissimilar from the healthy population for direct comparisons (Table 1; Table 2: *Unmatched Full Sample* column). Therefore, a Greedy Propensity Score Matching (PSM) algorithm(34, 35) was used to identify a comparable group of individuals from the healthy control pool. We used 1:1 matching without replacement(36) based on propensity scores generated by a logistic model designed to estimate the probability of having GD using the 31 Elixhauser co-morbidities and available demographic variables. In this way, we were able to identify 17,472 matched pairs for GD (Table 1; Table 2: *Matched Full Sample* column). To assess any differences in risk associated with race, ethnicity and/or gender we stratified the full sample into six race/ethnicity/gender-specific subgroups and re-ran the PSM algorithm. This resulted in the identification of 3,701 matched pairs for males, 13,722 for females, 14,558 for Whites, 1,742 for Blacks, 210 for Hispanics and 835 for individuals of other races. All analysis in this study is repeated for each of these subgroups.

The standardized difference (**Δ**_*s*_) (37) was used to assess the inter-group differences before and after PSM. The standardized difference is not affected by differences in sample size and has the benefit of being relatable to two other measures of association, the Pearson correlation coefficient for continuous and the phi coefficient for dichotomous variables(38, 39). We used the criterion of |**Δ**_*s*_ | ≤ 10 % to reduce the inter-group differences to a level sufficient for further analysis. Using this criterion, we judged that the PSM algorithm successfully reduced the inter-group differences to a level sufficient for further analysis (Table 2). There were some exceptions. The baseline ages for the GD group occurred 11.48 (Full sample), 13.51 (Female sample) and 15.05 (White sample) percentage points (pp) earlier on average than in their PSMed counterparts. None of these differences were greater than 1 year in real terms and this difference in age was explicitly addressed by the way we utilized age in our survival analysis models. The baseline dates for the GD group also occurred 10.49pp (Hispanic sample) to 20.73pp (White sample) earlier than those of their PSMed counterparts. However, none of these were greater than 2 years, and most under one year, in real terms. Individuals with GD were 12.25pp (Black sample) to 19.58pp (Male sample) percentage points less likely to be dual eligible than their PSMed counterparts. There were also some minor differences in the rates of uncomplicated diabetes in the White, Black, and Hispanic samples, but these were quite close to the 10-percentage point mark. Finally, the Hispanic subgroup (the smallest in terms of available controls for matching individuals with GD) demonstrated lower levels of other neurological disorders, diabetes with complications, lymphoma, and coagulopathy. Given the extent of the initial differences that were overcome by the PSM process, the extensive list of individual conditions used to de ne baseline health status, and the relatively small size of the control pool for some subgroups (e.g., Hispanics) we judged that these limitations were not detrimental to the study.

Survival analysis was done using two methods: the Cox proportional hazards model and the Fine-Gray competing risk model(40) with death as the competing risk. In both cases, age, the most important non-genetic risk factor for AD, was included non-parametrically as a time-scale variable. Thus, the partial likelihood is maximized for individuals with the same value of the time scale variable. Therefore, the effects of age in the model are accounted for non-parametrically and, in a certain sense, exactly. The only covariate explicitly included in the model was membership in the GD group. The PSM matching ensures that the GD and non-GD groups were nearly identical in terms of all other covariates at baseline. All analysis was done using SAS 9.4 software (Cary, NC: SAS Institute Inc.) after obtaining permission from the Duke University IRB.

## 3.0. Results

**Figure 1:**
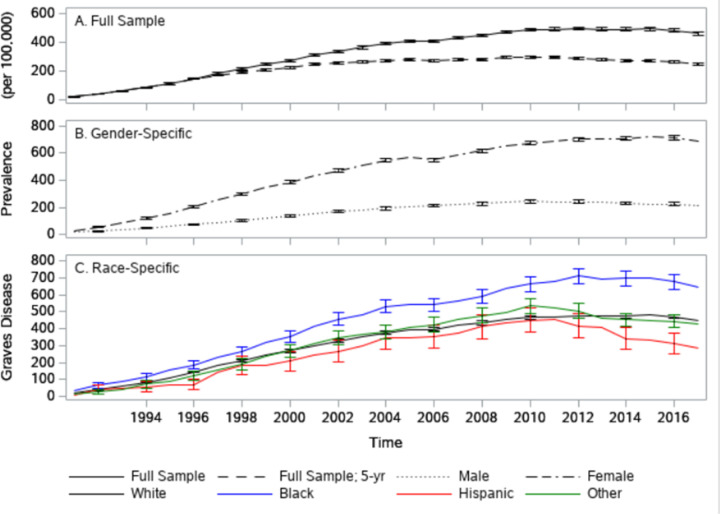
Trends in Graves Disease Prevalence Trend in the prevalence of individuals ever to be diagnosed with Graves Disease (per 100,000). Full sample, Graves Disease an absorbing state (Panel A: black solid line); Full sample, Graves Disease in remission after 5 years (Panel A: black dashed line); Males (Panel B: black dotted line), Females (Panel B: black dot-dash line), White (Panel C: black solid line), Black (Panel C: blue solid line), Hispanic (Panel C: red solid line), other (Panel C: green solid line).

The total, 65 + age-adjusted prevalence of GD grew over the study period reaching a maximum of 560 per 100,000 in 2012 (Fig. 1; Panel A). Note that the nature of GD (e.g., need for long-term treatment; high recurrence and low remission rates), combined with the low accuracy of identifying remission from administrative health data led to the decision to treat GD as a permanent condition. Therefore, the prevalence levels are likely to be overestimated. Making a counterfactual assumption, that all instances of GD are cured over 5 years, the initial estimates fall sharply (Fig. 1; Panel A). As expected, (Fig. 1; Panel B) the prevalence of GD is significantly higher in females (maximum of 721 per 100,000 in 2015) than in males (maximum of 244 per 100,000 in 2010). Blacks (Fig. 1; Panel C) have the highest prevalence of GD among all races (maximum of 709 per 100,000 in 2012) and this difference is statistically significant from all other groups from 2000 onwards. In contrast, Hispanics have the lowest prevalence of GD (maximum of 453 per 100,000 in 2011). However, these differences are not statistically different from other non-Black races/ethnicities until 2014.

**Figure 2:**
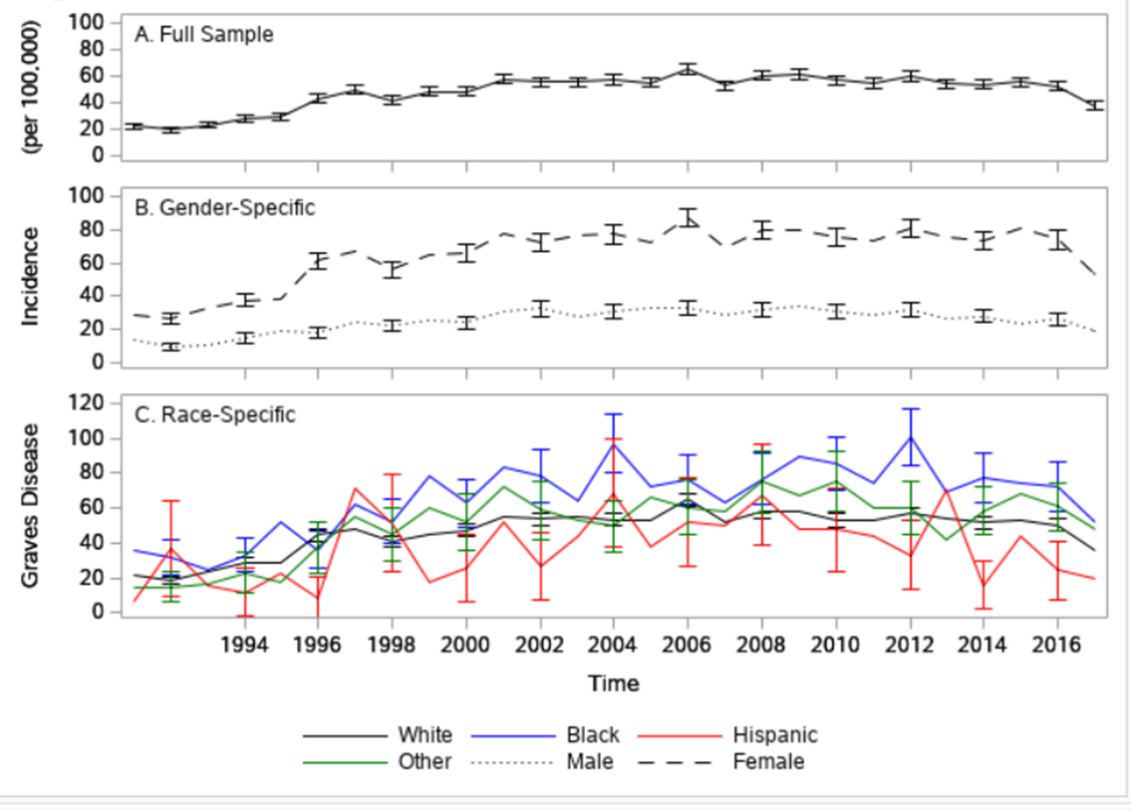
Trends in Graves Disease Incidence Trend in the incidence of Graves Disease (per 100,000). Full sample (Panel A: black solid line) Males (Panel B: black dotted line), Females (Panel B: black dash line), White (Panel C: black solid line), Black (Panel C: blue solid line), Hispanic (Panel C: red solid line), other (Panel C: green solid line).

The total 65 + age adjusted incidence of GD, although subject to some fluctuations, is fairly constant with a maximum of 65 per 100,000 in 2006 (Fig. 2; Panel A). Like with prevalence, GD incidence (Fig. 2; Panel A) in females (maximum of 87 per 100,000 in 2006) is significantly higher than that of males (maximum of 33 in 100,000 in 2006). However, unlike prevalence, no strong race/ethnicity-related patterns in incidence can be observed (Fig. 2; Panel C). Blacks appear to have the highest incidence rates (maximum of 100 per 100,000 in 2012) and Hispanics the lowest. However, the race/ethnicity-specific confidence intervals overlap. Only in 2012, can we say with any statistical confidence, that Black incidence rates of GD are higher than those of Hispanics.

Survival analysis results are presented in Table 3. The Cox model shows that in a PSM sample, the presence of Graves disease is associated with higher risk of AD in the full sample (Hazard Ratio [HR]:1.15; 95% Confidence Interval [CI]:1.07–1.23), Males (HR:1.19; CI:1.01–1.41), Females (HR:1.09; CI:1.02–1.18), Whites (HR:1.13; CI:1.04–1.20), and Blacks (HR:1.33; CI:1.04–1.20). Although the effects of GD on AD risk in Hispanics and individuals of other races is insignificant, the sign of the HR is consistent with the results obtained for other subgroups and the CI overlaps those of the significant results. The results of the competing risk model, although lower on average, are highly consistent with those of the traditional Cox model. The increased risk of AD onset associated with GD was significantly higher in the general 65 + population, Males, Whites, and Blacks. The effect direction and confidence intervals of the subgroups for which a statistically significant effect could not be identified was consistent with the effects identified in all other study subgroups. No race/ethnicity/gender-related disparities in the effect of GD on AD could be observed as the CI for all study subgroups overlap.

## 4.0. Discussion

In this paper we found that the presence of GD is associated with a significantly higher risk of clinical AD. This finding is in line with earlier reports of the increased risk of AD/dementia in individuals with hyperthyroidism (26, 30, 41). A potential mechanism connecting GD (and related hyperthyroidism) to AD may involve shared etiological factors between the two diseases, such as viral infections and compromised/aberrant immunity, which may themselves contribute to the development of both GD and AD (31, 42–46). However, evidence backing any single mechanism is still inconclusive, and some studies failed to identify the association between hyperthyroidism and cognitive function or dementia (47). Furthermore, earlier research found that both low and high thyrotropin (a pituitary hormone that stimulates the production of thyroid hormones) could be associated with increased risk of AD (28), suggesting possibility of U-shaped relationship. One potential reason for these findings might be that the role of metabolism could be contextual (48, 49). For example, slowdowns in metabolism might promote dementia through declining rates of information processing or by impairing the resilience of the body to adverse health events, such as infections, through delayed immune responses, slower healing, and longer recovery time (49). On the other hand, it may also slow some aging-related changes in the body and be beneficial in the long term by, for example, reducing the rate of damage accumulation in the brain(48, 49).

This study has several strengths. First, it was conducted after equalizing the GD and non-GD groups across a wide range of demographic and health-related conditions. This is vital as the GD group was shown to be statistically different from the non-GD population across many health-related conditions, including AD risk-related diseases. Second, it is based on data nationally representative of the 65 + population with follow-up periods of up to 27 years. This provides the study with a relatively large group of individuals with a clinical diagnosis of GD even in smaller population strata. This is also important as both GD and AD are rare outcomes, making identification of results generalizable to the U.S. population challenging. However, this study also has limitations. It is based on administrative claims data designed for billing purposes, not medical research. Therefore, valuable information, such as the results of laboratory analyses, is not available. However, the disease ascertainment algorithm used is designed to reduce the impact of tentative and mistaken diagnoses (essentially we are assuming that if the laboratory results were not consistent with a diagnosis of GD, then it would not be listed as a diagnosis on the second visit) and our estimates of the extra risk of AD onset associated with GD are highly consistent with the estimates of at least one study based on biological measurements(33).

Similarly, AD, in the context of our data, represents a clinical diagnosis of *possible/probable Alzheimer’s Disease dementia*, and may not reflect the exact etiology of the individual’s actual condition. AD is often mistaken for other conditions and, often co-exists with other types of dementia making its diagnosis before autopsy di cult even at the bedside. The sample size for some race/ethnicity-related strata (especially Hispanics) in this study is too small to generalize with confidence. Even though the study is nationally representative, and the sample size reflects the true situation as capturable by this dataset, additional studies with a focus on oversampling minority groups are warranted. Finally, we were not able to differentiate between the effects of treated/controlled GD and situations where disease management is proving a challenge or of any additional risk associated with alternative types of GD treatment. This is an important avenue of future research as studies have shown that for some chronic health conditions related to AD risk, aggressive management of the risk-related disease acts to reduce the associated AD risk as well(50).

## 5.0. Conclusion

In the absence of proven treatments to slow and/or reverse the neurodegenerative effects of AD, identification of modifiable risk factors is an important aspect of controlling the burden imposed by this disease on an increasing number of older U.S. adults. In this study, we provide evidence for GD as one such risk factor. Although the exact biological mechanisms potentially linking the two conditions are unclear and focused studies of race/ethnicity-specific subgroups as well as the replication of these findings on datasets with available biomarkers and laboratory test results are needed, our findings suggest that GD prevention and treatment may contribute to reducing the burden of AD in U.S. older adults.

## Figures and Tables

**Table 1:** Summary Statistics

	Full Sample	Matched Sample
*Baseline Age*	72.02 (6.30)	75.03 (7.91)
*Male*	0.41 (0.49)	0.23 (0.42)
*White*	0.86 (0.35)	0.82 (0.39)
*Black*	0.08 (0.27)	0.11 (0.31)
*Hispanic*	0.02 (0.12)	0.02 (0.13)
*Other*	0.04 (0.20)	0.05 (0.23)
*Ever Dual Eligible*	0.19 (0.39)	0.22 (0.42)
*Yearly Trend (2000 = 0)*	−1.31 (7.72)	−4.42 (7.38)
*Congestive heart failure*	0.06 (0.24)	0.19 (0.39)
*Cardiac arrhythmias*	0.10 (0.30)	0.32 (0.47)
*Valvular disease*	0.04 (0.19)	0.14 (0.35)
*Pulmonary circulation Disorders*	0.01 (0.10)	0.04 (0.20)
*Peripheral vascular disorders*	0.06 (0.23)	0.16 (0.37)
*Hypertension, uncomplicated*	0.38 (0.49)	0.66 (0.47)
*Hypertension, complicated*	0.04 (0.19)	0.13 (0.34)
*Paralysis*	0.01 (0.09)	0.02 (0.14)
*Other neurological disorders*	0.02 (0.15)	0.06 (0.24)
*Chronic pulmonary disease*	0.11 (0.32)	0.25 (0.44)
*Diabetes, uncomplicated*	0.14 (0.35)	0.25 (0.44)
*Diabetes, complicated*	0.04 (0.19)	0.09 (0.29)
*Hypothyroidism*	0.07 (0.26)	0.42 (0.49)
*Renal failure*	0.02 (0.14)	0.06 (0.24)
*Liver disease*	0.01 (0.11)	0.04 (0.20)
*Peptic ulcer disease excluding bleeding*	0.01 (0.11)	0.03 (0.18)
*AIDS/H1V*	< 0.01 (0.02)	< 0.01 (0.02)
*Lymphoma*	< 0.01 (0.07)	0.01 (0.11)
*Metastatic cancer*	0.01 (0.09)	0.02 (0.14)
*Solid tumor without metastasis*	0.07 (0.26)	0.15 (0.36)
*Rheumatoid arthritis/ collagen vascular diseases*	0.03 (0.17)	0.09 (0.29)
*Coagulopathy*	0.01 (0.12)	0.05 (0.22)
*Obesity*	0.02 (0.14)	0.05 (0.21)
*Weight loss*	0.01 (0.12)	0.10 (0.30)
*Fluid and electrolyte disorders*	0.04 (0.20)	0.16 (0.37)
*Blood loss anemia*	0.01 (0.07)	0.02 (0.15)
*Deficiency anemia*	0.03 (0.16)	0.10 (0.31)
*Alcohol abuse*	< 0.01 (0.07)	0.01 (0.09)
*Drug abuse*	< 0.01 (0.04)	< 0.01 (0.07)
*Psychoses*	0.01 (0.10)	0.02 (0.14)
*Depression*	0.05 (0.21)	0.13 (0.33)
**N**	3,109,022	34,944
**N Graves Disease**	17,505 (0.56%)	17,472 (50.00%)
**N Alzheimer’s Disease**	300,635 (9.67%)	3,479 (10.00%)
**N Dead**	1,287,014 (41.40%)	14,513 (41.47%)

Note: Numbers presented are sample means with standard deviations in parentheses.

**Table 2:** Propensity Score Matching Quality: Standardized Differences Between the Graves Disease and No Graves Disease Groups

	*Unmatched*	Propensity Score Matched Samples						
	*Full Sample*	*Full*	*Male*	*Female*	*White*	*Black*	*Hispanic*	*Other*
*Baseline Age*	**38.44**	**−11.48**	*−8.96*	**−13.51**	**−15.05**	−8.72	3.11	−4.57
*Male*	**−43.76**	−6.33	*N.A*	*N.A*.	−4.43	−5.55	−3.13	−4.79
*White*	−6.69	8.46	15.03	9.04	*N.A*.			
*Black*	8.60	−3.80	−8.11	−4.04				
*Hispanic*	−2.95	−6.19	−9.64	−6.14				
*Other*	2.69	−4.96	−6.10	−5.68				
*Ever Dual Eligible*	1.06	**−15.33**	**−19.58**	**−18.19**	**−13.44**	**−12.25**	−5.77	−7.70
*Yearly Trend (2000 = 0)*	**−50.80**	**−14.70**	**−26.53**	**−19.59**	**−20.73**	−9.30	**−10.49**	**−11.27**
*Congestive heart failure*	**36.65**	−7.64	−8.62	−8.50	−7.71	−3.02	−6.49	0.67
*Cardiac arrhythmias*	**55.43**	−4.54	−8.60	−7.07	−8.41	−0.64	−4.11	<0.01
*Valvular disease*	**35.32**	−3.87	−5.10	−4.73	−6.06	−1.43	5.98	−2.36
*Pulmonary circulation Disorders*	**19.44**	−2.33	−3.77	−2.37	−1.90	0.78	<0.01	4.18
*Peripheral vascular disorders*	**31.24**	−8.50	−5.93	−5.42	−7.06	−7.55	−3.55	−5.41
*Hypertension, uncomplicated*	**54.77**	−6.62	−4.31	−5.86	−6.78	−6.01	7.15	−7.02
*Hypertension, complicated*	**31.80**	−5.21	−4.08	−4.74	−4.57	−2.80	7.26	−6.19
*Paralysis*	8.03	−2.92	−1.63	−3.27	−2.88	−4.41	<0.01	<0.01
*Other neurological disorders*	**16.36**	−5.24	−6.68	−6.46	−4.17	−2.71	**−14.36**	2.82
*Chronic pulmonary disease*	**34.78**	−6.18	−7.16	−5.98	−9.09	−3.70	−6.93	−4.20
*Diabetes, uncomplicated*	**25.34**	−7.70	−7.16	**−10.97**	**−11.09**	**−10.64**	−4.00	−6.15
*Diabetes, complicated*	**21.26**	−5.60	−4.59	−6.25	−6.48	−9.08	**−11.30**	−1.50
*Hypothyroidism*	**91.81**	4.99	1.28	6.58	6.07	1.07	−3.89	−0.25
*Renal failure*	**21.75**	−2.62	−5.71	−3.45	−3.65	−3.54	3.69	1.08
*Liver disease*	**16.84**	−2.32	−4.01	−3.64	−2.19	−4.47	−5.62	−3.55
*Peptic ulcer disease excluding bleeding*	**13.36**	−1.14	−3.08	−4.40	−2.82	3.90	−1.77	3.49
*AIDS/H1V*	−0.07	−0.30	−1.34	N.A.	−1.17	1.96	<0.01	<0.01
*Lymphoma*	6.31	−2.17	−1.65	−1.38	−3.04	0.59	**−13.17**	2.83
*Metastatic cancer*	9.89	−1.19	−4.36	−1.41	−3.49	−1.83	N.A.	−0.92
*Solid tumor without metastasis*	**25.58**	−2.89	−4.25	−3.64	−3.59	−2.94	0.00	−2.86
*Rheumatoid arthritis/ collagen vascular diseases*	**25.17**	−4.13	−4.04	−2.77	−2.87	−3.57	−3.10	−0.47
*Coagulopathy*	**21.12**	−2.05	−3.81	−3.43	−3.62	−2.00	**10.60**	−0.66
*Obesity*	**12.91**	−3.81	−1.97	−4.25	−4.45	−5.78	−5.16	−1.43
*Weight loss*	**37.61**	−0.78	−4.41	−2.99	−1.52	−2.18	−1.64	0.00
*Fluid and electrolyte disorders*	**36.95**	−4.88	−5.75	−6.32	−5.71	−4.38	7.26	0.36
*Blood loss anemia*	**15.29**	−0.65	−1.39	−3.18	−0.96	−2.89	0.00	0.95
*Deficiency anemia*	**30.91**	−2.73	−3.06	−6.18	−3.20	−5.16	−2.99	−6.13
*Alcohol abuse*	1.94	−2.87	−5.46	−2.68	−3.06	−0.63	−5.64	1.63
*Drug abuse*	5.52	−1.62	0.00	0.60	−1.59	<0.01	9.76	<0.01
*Psychoses*	7.57	−3.34	−1.68	−6.07	−3.95	−2.63	−3.71	−2.10
*Depression*	**25.58**	−5.98	−4.55	−5.80	−7.14	−6.09	4.23	0.87
**N**	3,109,022	34,944	7,402	27,444	29,116	3,484	420	1,670
**N Graves Disease**	17,505	17,472	3,701	13,722	14,558	1,742	210	835
**N Alzheimer’s Disease**	300,635	3,479	599	2,995	2,912	390	52	162

Note: Numbers presented are standardized differences; Standardized differences with an absolute value greater than 10 are in bold.

**Table 3:** Survival Analysis Results

	Full Sample	Male	Female	White	Black	Hispanic	Other
*Cox*	**1.15** **	**1.19** **	**1.09** **	**1.13** **	**1.33** **	1.11	1.25
	**[1.07–1.23]**	**[1.01–1.41]**	**[1.02–1.18]**	**[1.04–1.20]**	**[1.08–1.63]**	[0.64–1.95]	[0.92–1.71]
*Competing Risk*	**1.14** **	**1.19** **	1.07	**1.11** **	**1.31** **	1.05	1.19
	**[1.07–1.22]**	**[1.02–1.41]**	[0.99–1.54]	**[1.03–1.19]**	**[1.07–1.61]**	[0.60–1.83]	[0.88–1.61]
*N Matched Pairs*	17,472	3,701	13,722	14,558	1,742	210	835

* significant at the alpha = 0.05 level;

** significant at the alpha = 0.01 level

## Data Availability

This article is based on restricted data, not publicly available, but can be obtained from the Centers for Medicare and Medicaid Services.

## List Of Abbreviations

AD: Alzheimer’s Disease
CI: 95% Confidence interval
CMS: Centers for Medicare and Medicaid Services
GD: Graves Disease
HR: Hazard Ratio
ICD: International Classification of Disease
PSM: Propensity Score Matching
